# Supplementary material for: Use of artificial intelligence to recover mandibular morphology after disease
Source: Sci Rep. 2020 Oct 2;10:16431. doi: 10.1038/s41598-020-73394-5 (PMC7532179; doi:10.1038/s41598-020-73394-5)
Supplement: Supplementary file 2 — Supplementary Legend. [file 41598_2020_73394_MOESM2_ESM.docx]

**Video captions**

This article comes with a video summary to quickly explain the content of the article and more intuitive and concrete results. The video consists of 3 steps. Step 1 shows the training process of the generator. The 9 images in the video are samples from real training. This is the result of the generator generation of 9 constant input parameters during the 0-45-hour training process. This shows the progress that the generator has made in training. In the latter part of step 1, the background image shows the other generation results using the trained generator in the case of random input, showing the diversity of the generated results. Step 2 shows the 2D image completion process, first marking the lesion and then using the CTGAN algorithm to complete. In this step, the completion of a CBCT continuous layer in a virtual environment is described in a specific clinical case. Step 3 shows the 3D image completion process. This mandible comes from a patient whose mandibular defect spans the midline and has a large area. First, reduce the resolution of the CBCT data and then create a 3D mask marker defect area. The 3D completion of the jaw was completed by an algorithm. In this step, the original 3D model of the CBCT in this case is given, and the multiangle topography of the low-resolution model and the complemented model before completion is given.
